# La crosse aortique droite avec aorte descendante gauche: une cause rare de dysphagie

**DOI:** 10.11604/pamj.2020.36.17.17206

**Published:** 2020-05-13

**Authors:** David Douglas Banga Nkomo, Louis Joss Bitang A Mafok

**Affiliations:** 1 Centre des Urgences de Yaoundé, Yaoundé, Cameroun

**Keywords:** Crosse aortique droite, aorte descendante gauche, dysphagie, Right aortic arch, left descending aorta, dysphagia

## Abstract

Aortic abnormalities are a rare cause of dysphagia in children, adolescents, young adults and in the elderly. In these cases, vascular abnormalities commonly include aberrant right subclavian artery, left subclavian artery originating from the right aortic arch and, more rarely, from the right aortic arch with thoracic aorta on the left. Dysphagia, also known as “dysphagia lusoria”, is due to the compression of the esophagus caused by one or several congenital vascular abnormalities and can occur in childhood or later in adulthood. Upper gastrointestinal series show extrinsic compression of the esophagus and thoracic angiography (scanner or magnetic resonance imaging (MRI)) is used to confirm the diagnosis. In a few cases, surgical treatment is often required. We here report the case of a 30-year old female patient, with no previous medical or surgical history, presenting with dysphagia to solid foods of recent onset, without other associated symptom. Physical examination showed good general condition. The patient underwent upper gastrointestinal series revealing extrinsic esophageal compression, then chest X-ray and chest MRI showing right aortic arch with left descending aorta, without situs inversus. Finally, echocardiography was performed which excluded associated congenital heart disease. Outcome was marked by spontaneous regression of dysphagia.

## Image en médecine

Les anomalies aortiques constituent une étiologie rare des dysphagies chez les grands enfants, les adolescents, les adultes jeunes et les personnes âgées. Dans ces cas, l´anomalie vasculaire en cause est, très souvent une artère sous-clavière droite aberrante, une artère sous-clavière gauche naissant d´une crosse aortique droite et plus rarement une crosse aortique à droite avec une aorte thoracique descendante à gauche. Cette dysphagie, appelée “dysphagia lusoria”, due à la compression de l´œsophage par une ou plusieurs anomalies vasculaires congénitales, peut apparaitre dans l´enfance ou plutard à l´âge adulte. Le transit œsogastroduodénal révèle une compression extrinsèque de l´œsophage et l´angiographie (scanner ou imagerie par résonnance magnétique (IRM)) thoracique confirme le diagnostic. Dans quelques cas, un traitement chirurgical est souvent requis. Nous rapportons le cas d´une patiente de 30 ans, sans antécédent médical ou chirurgical contributif, venue consulter pour une dysphagie aux solides, d´installation récente, sans autre symptôme associé. Elle avait un bon état général, et son examen physique était normal. Dans le cadre de l´exploration de cette dysphagie, la patiente réalisera d´abord un transit œsogastroduodénal qui révèlera une compression œsophagienne extrinsèque, puis secondairement une radiographie du thorax et une imagerie par résonnance magnétique thoracique qui mettront en évidence une crosse aortique droite avec une aorte descendante gauche, sans situs inversus. L´échographie doppler cardiaque qui sera réalisée en dernier éliminera une cardiopathie congénitale associée. L´évolution a été marquée par la régression de façon spontanée de la dysphagie.

**Figure 1:**
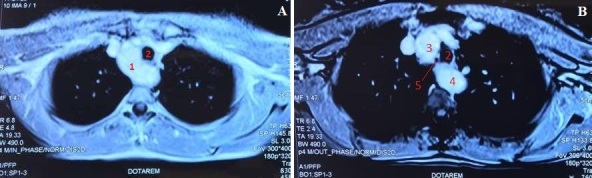
Coupe IRM thoracique d'une crosse aortique droite avec une aorte descendante à gauche; A) 1: crosse de l´aorte; 2: trachée; B) 3: aorte ascendante; 4: aorte descendante; 5: œsophage

